# Optimal Depression Screening Interval Among Students in Higher Education

**DOI:** 10.1192/j.eurpsy.2026.10777

**Published:** 2026-07-31

**Authors:** M. Hayden, S. Hartman, R. Ferrara, R. Farah

**Affiliations:** Student Health and Wellness, University of Virginia, Charlottesville, VA, United States

## Abstract

**Introduction:**

Despite the high burden of depression among young adults enrolled in higher education, the ideal frequency for depression screening in this population is unknown. Current guidelines for depression screening from the U.S. Preventive Services Task Force for adults and adolescents do not provide a specific screening frequency due to lack of evidence.

**Objectives:**

To identify the ideal interval between depression screenings at medical visits among university students.

**Methods:**

We analyzed data from a 2-year period (04/24/2022 through 04/24/2024) of students who were offered a depression screen (PHQ2) during an outpatient medical visit at a large, 4-year public university Student Health Center (SHC). During the study period, the PHQ2 was offered at every provider visit in the medical clinic; patients could decline screening. For scores ≥2, the PHQ9 was subsequently administered.

A negative depression screening was defined as either a negative PHQ2, or a positive PHQ2 and a negative PHQ9 (score ≤4). A positive depression screening was defined as a PHQ9 score >4; mild=score 4-9, moderate=score 10-14, severe=score >14.

The time difference in days was calculated for each unique patient by identifying their first positive depression screening and their immediately previous negative screening. The time difference for all patients was summarized as median and interquartile range.

We utilized the Student Health Research Database, an IRB-approved de-identified dataset linking electronic medical record data with university student enrollment data, to evaluate all PHQ2 and PHQ9 responses.

**Results:**

During the study period, 17,721 unique students were offered a depression screen. Of these, 739 unique students had a positive depression screen following an initial negative depression screen (10.9%): 397 students (54%) with score of mild, 209 students (28%) with a score of moderate, and 132 students (18%) with a score of severe.

The overall median interval between a negative depression screen and positive PHQ9 score was 77 days (Interquartile range: 28-165).

**Conclusions:**

Providing guidance to college health clinicians on the frequency of depression screening allows for optimal identification and management of struggling students and consistency in practice among SHCs. In 2022, 31% of 18–24-year-olds in the US were enrolled in a 4-year college or university (Integrated Postsecondary Education Data System, accessed July 2025). For these students, the SHC serves as a highly accessible option for medical care and most universities offer access to mental health care, either on-site or through referral to community or telehealth options. In this study, 2-3 months was identified as the ideal interval between depression screenings. Such screenings can be performed during any encounter with the student; the clinician should not wait for a preventive care visit.

**Disclosure of Interest:**

None Declared

